# Elevated Serum Soluble Tim-3 in Primary Biliary Cholangitis: Lack of Correlation with Cytokines, Chemokines, and Clinical Parameters

**DOI:** 10.5152/tjg.2025.24520

**Published:** 2025-08-25

**Authors:** Jiamin Xu, Haitao Ma, Futao Dang, Hua Lin, Chenrui Zhang, Qian Wang, Xu Tan, Xian Yang, Jingyi Zhang, Weimin Bao, Yingmei Tang

**Affiliations:** 1 Department of Gastroenterology, The Second Affiliated Hospital of Kunming Medical University, Yunnan, China; 2 Department of Clinical Laboratory, Yunnan Molecular Diagnostic Center, The Second Affiliated Hospital of Kunming Medical University, Yunnan, China; 3 Department of Colorectal Surgery, The First People’s Hospital, Yunnan, China

**Keywords:** Chemokine, cytokine, galectin-9, primary biliary cholangitis, soluble Tim-3

## Abstract

**Background/Aims:**

Soluble Tim-3 (sTim-3) has been implicated in primary biliary cholangitis (PBC), an autoimmune liver disease, though its clinical significance remains unclear. This study aimed to evaluate the associations between sTim-3, Galectin-9, and cytokines in PBC, as well as their potential prognostic utility.

**Materials and Methods:**

A total of 55 PBC patients were enrolled (45 without overlapping conditions) and serum levels of sTim-3, Galectin-9, and 18 cytokines/chemokines were measured. Disease severity was assessed using the model for end-stage liver disease (MELD), MELD-Na, and Mayo risk score (MRS) 1994, alongside fibrosis-4 (FIB-4) index and monocyte-lymphocyte ratio (MLR). Patients were stratified by fibrosis stage, cirrhosis status, Child-Pugh score, and treatment duration, with intergroup parameter comparisons performed. The least absolute shrinkage and selection operator regression identified potential risk factors for MRS1994, followed by multivariate linear regression analysis.

**Results:**

Compared to healthy controls, PBC patients exhibited elevated sTim-3 and reduced Galectin-9, though neither biomarker correlated with clinical parameters. Advanced disease stages were associated with increased MLR, interferon-gamma (IFN-γ), interleukin (IL)-6, IL-8, C-C motif chemokine ligand 3 (CCL3), CCL20, and C-X3-C motif chemokine ligand 1 (CX3CL1). The MELD/MELD-Na scores strongly correlated with IL-6, TNF-α, IFN-γ, CCL3, and CCL20, while IL-6 and CX3CL1 linked to FIB-4 index. Multivariate analysis identified MLR, albumin/globulin (A/G) ratio, TNF-α, IL-6, and CX3CL1 as independent predictors of MRS1994.

**Conclusion:**

Although sTim-3 and Galectin-9 dysregulation lacked direct clinical relevance, MLR, A/G ratio, cirrhosis status, and inflammatory markers (TNF-α, IL-6, CX3CL1) emerged as robust predictors of disease severity (MELD) and prognosis (MRS1994), highlighting their potential for non-invasive risk stratification in PBC.

**Main Points**

- Despite observed dysregulation of soluble Tim-3 and Galectin-9 in PBC patients, these biomarkers showed no significant association with clinical disease parameters.
- The MLR, A/G ratio, TNF-α, interleukin-6, and CX3CL1 demonstrate predictive value for disease severity and prognosis in PBC.

## Introduction

Primary biliary cholangitis (PBC) is an autoimmune liver disease characterized by nonsuppurative destructive cholangitis, the presence of anti-mitochondrial antibody M2 (AMA-M2) or PBC-specific antinuclear antibodies, and chronic progressive cholestasis.^1^ The global prevalence of PBC is highest in North America, followed by Europe and Asia-Pacific, with steady increases observed across all geographical areas.^2^ Up to 40% of PBC patients exhibit a suboptimal response to first-line ursodeoxycholic acid (UDCA) therapy, while even UDCA responders remain at risk of adverse clinical outcomes.^3^^,^^4^ Immune dysregulation is a hallmark feature of PBC, in which cytokines, chemokines, and immune checkpoints play critical pathological roles in driving autoimmunity.^5^

Dysregulation of immune checkpoints, including T-cell immunoglobulin and mucin domain-containing molecule 3 (Tim-3), is critically implicated in the pathogenesis of various autoimmune diseases.^6^ The involvement of the Tim-3/Galectin-9 pathway in a murine model of PBC was identified in previous work.^7^ Emerging evidence suggests that elevated levels of soluble immune checkpoints are associated with disease severity and prognosis in autoimmune conditions.^8^ A study has demonstrated that higher circulating levels of soluble Tim-3 (sTim-3) may serve as a potential biomarker for distinguishing autoimmune hepatitis (AIH) from PBC.^9^ Several soluble immune checkpoints, including sTim-3, have been reported to be significantly elevated in PBC and linked to disease staging.^10^ Notably, soluble immune checkpoints can block immune checkpoint signaling, leading to increased production of pro-inflammatory cytokines and chemokines.^8^ To date, the relationship between sTim-3 and cytokines/chemokines, as well as its broader clinical significance in PBC patients, remains incompletely understood.

Monocyte-related variables, including the monocyte-lymphocyte ratio (MLR), may serve as potential biomarkers for evaluating the efficacy and safety of immune checkpoint inhibitors.^11^ While Tim-3 plays a crucial regulatory role in the immune microenvironment,^12^ its potential indirect effects on MLR and albumin/globulin (A/G) ratio remain to be elucidated. This study measured serum levels of sTim-3, Galectin-9, and 18 cytokines/chemokines additionally, along with conventional blood biochemical parameters, including MLR and other non-invasive indicators. Subgroup analyses were performed to identify differences and explore correlations among these variables. Furthermore, their potential as prognostic markers for liver fibrosis severity and clinical outcomes in PBC was investigated.

## Materials and Methods

### Study Subjects

**Figure 1.**
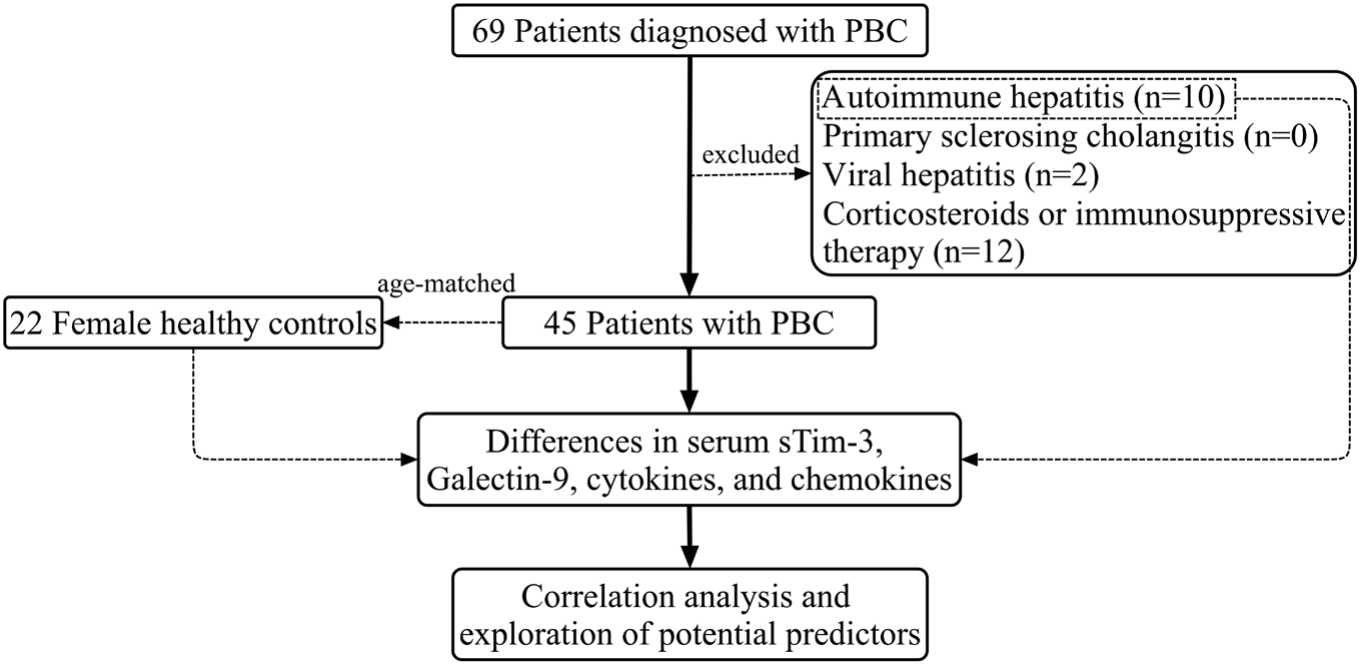
Study enrollment flowchart. PBC, primary biliary cholangitis; sTim-3, soluble T-cell immunoglobulin and mucin domain 3.

Patients diagnosed with PBC based on established criteria through liver biopsy and laboratory parameters were included in the study. Patients with concurrent AIH (PBC-AIH OS) were included solely for intergroup comparisons of serum sTim-3, Galectin-9, cytokines, and chemokines with healthy controls (HC) and PBC groups and were subsequently excluded from further statistical analyses. Exclusion criteria included: (1) other autoimmune diseases (including but not limited to Hashimoto’s thyroiditis, systemic sclerosis, and systemic lupus erythematosus); (2) hepatobiliary disorders (primary sclerosing cholangitis, viral hepatitis); and (3) any conditions requiring corticosteroid or immunosuppressive therapy. The PBC patients were stratified by stage of fibrosis, cirrhosis status, Child-Pugh score, and treatment duration for parameter comparisons. Figure 1 presents a flowchart of the inclusion and exclusion processes. All participants were recruited from the hospital between November 2020 and May 2021. Healthy controls consisted of age-matched female individuals recruited from the hospital’s physical examination center. The study protocol was approved by the ethic committee of the the Second Affiliated Hospital of Kunming Medical University (Date: November 9, 2020; Approval no: PJ-2020-96 and Date: June 11, 2021 Approval no: PJ-2021-73), and all participants provided written and verbal informed consent.

### Serum Samples and Measurements

Following routine biochemical tests, serum samples were collected from the clinical laboratory, and stored at −80°C. Serum concentrations of cytokines (interferon-gamma (IFN-γ), TNF-α, interleukin (IL)-1β, IL-2, IL-6, IL-8, IL-10, IL-12), and chemokines (CCL2, chemokine ligand 3 (CCL3), CCL4, CCL19, CCL20, CX3CL1, CXCL9, CXCL10, CXCL11, CXCL16) were quantified using the Human Magnetic Luminex Screening Assay (LXSAHM-18, R&D Systems, Minneapolis, MN, USA) on a Luminex® system (MAGPIX® with xPONENT software, Luminex, Austin, TX, USA) following the manufacturer’s protocol.

Serum levels of sTim-3 and Galectin-9 were measured using enzyme-linked immunosorbent assay (ELISA) with human TIM-3 (BMS2219, Invitrogen, Carlsbad, CA, USA) and GAL9 (EH0148, Fine test, Wuhan, Hubei, China) ELISA kits, respectively.

### Data Collection and Calculation

Collected clinical and laboratory data included age, gender, age at PBC diagnosis, liver histology, routine laboratory blood tests (complete blood count, liver and renal function tests), PBC-specific autoantibody status (AMA-M2, GP210, and SP100), and treatment details (medication, dosage, and duration). Based on Ludwig and Scheuer’s histologic classification,^13^^,^^14^ PBC patients were categorized as “Early stage” (histological stages I-II) or “Advanced stage” (histological stages III-IV).^15^^,^^16^ Patients were stratified by UDCA treatment duration (13-15 mg/kg/day orally) into 3 subgroups: treatment-naïve (0 months), <12 months, and ≥12 months. Non-invasive liver disease prognostic indicators were calculated, including (1) Fibrosis-4 (FIB-4): age (years) × AST (U/L)/(pcount (109/L) × √ALT (U/L)); (2) Model for End-Stage Liver Disease (MELD): 3.78 × ln (bilirubin [mg/dL]) + 11.2 × ln (INR) + 9.57 × ln (creatinine [mg/dL]) + 6.43; (3) MELD-Na: MELD + 1.32 × (135 − Na [mmol/L]) − 0.033 × MELD × (135 − Na [mmol/L]); (4) Child-Pugh score (incorporating bilirubin, albumin, INR/prothrombin time, ascites, and hepatic encephalopathy), with PBC-specific bilirubin thresholds: 1 point (≤68 μmol/L), 2 points (≤170 μmol/L)17; and (5) Mayo risk score (MRS) 1994 (MRS1994): 0.051 × age (years) + 1.209 × ln(bilirubin [mg/dL]) + 2.754 × ln (platelet count [10^9^/L]) + 0.675 × edema score + 3.304 × ln (albumin [g/dL]).^18^ Edema scoring: 0 (no history), 0.5 (present without diuretics or resolved with treatment), 1 (persistent despite diuretics).

### Statistical Analysis

Two Child-Pugh class C patients were reclassified as class B-C. For IL-10 and IL-12 measurements below the lower limit of detection (LLD) (1.17 pg/mL and 116.89 pg/mL, respectively), LLD values were imputed.^19^

Baseline characteristics are presented as percentages for categorical variables. Continuous variables are expressed as mean ± standard deviation (if normally distributed data) or medians with interquartile range (IQR) (if non-normally distributed data). Univariate analyses of categorical variables were performed using either the chi-square test or Fisher’s exact test, as appropriate. Normally distributed continuous variables were analyzed with Student’s *t*-test (for 2-groups comparisons) or 1-way ANOVA (for multiple groups), while non-normally distributed continuous variables were evaluated using the non-parametric Wilcoxon rank-sum test (2 groups) or Kruskal–Wallis test (multiple groups). Spearman’s rank correlation coefficient was utilized to examine the relationships between cytokine levels and clinical parameters.

Furthermore, linear regression analysis was implemented to evaluate the prognostic potential of immune-related variables. The multivariable linear regression model incorporated variables selected through a 2-step process: First, univariate analysis identified variables demonstrating significant differences in immune-related parameters that also showed moderate-to-strong correlations with MRS1994 (Spearman’s |r| ≥ 0.4). Second, these candidate variables underwent feature selection via the least absolute shrinkage and selection operator (LASSO) regression algorithm. The LASSO regression, executed using the glmnet package in R, optimized the linear regression model for MRS1994 prediction. Optimal tuning parameters (λ) were determined through 10-fold cross-validation, with the final variable subset selected based on mean squared error. Covariance analysis employing the F-test (from the car package) evaluated the statistical significance of selected variables in the linear model. The most influential LASSO-selected variables were subsequently incorporated into the final multivariable model. Statistical significance was defined as *P* < .05. All analyses were performed using R version 4.1.2 (R Foundation for Statistical Computing, Vienna, Austria) and OriginPro version 9.8, Learning Edition (OriginLab Corporation, Northampton, MA, USA).

## Results

### Characteristics of Subjects

The study enrolled 55 patients, including 45 with PBC and 10 with PBC-AIH OS. As detailed in Table 1, PBC-AIH OS patients demonstrated younger age at diagnosis (48 ± 13 years) compared to PBC-only patients (55 ± 11 years). The PBC-only patients were predominantly female (86.7%). Regarding PBC-specific autoantibodies, 83.7% were AMA-M2 positive, 37.2% were anti-gp210 positive, and 7.14% were anti-sp100 positive. Advanced disease stages predominated (80.0%), with cirrhosis present in 68.9% of PBC patients. Additionally, 40.0% were treatment-naïve at enrollment. Comprehensive laboratory parameters including FIB-4, MELD, MELD-Na, and MRS1994 scores are presented in Table 1.

### Elevated Circulating Monocyte and Reduced Albumin/Globulin Ratio in Advanced Primary Biliary Cholangitis Patients

**Figure 2.**
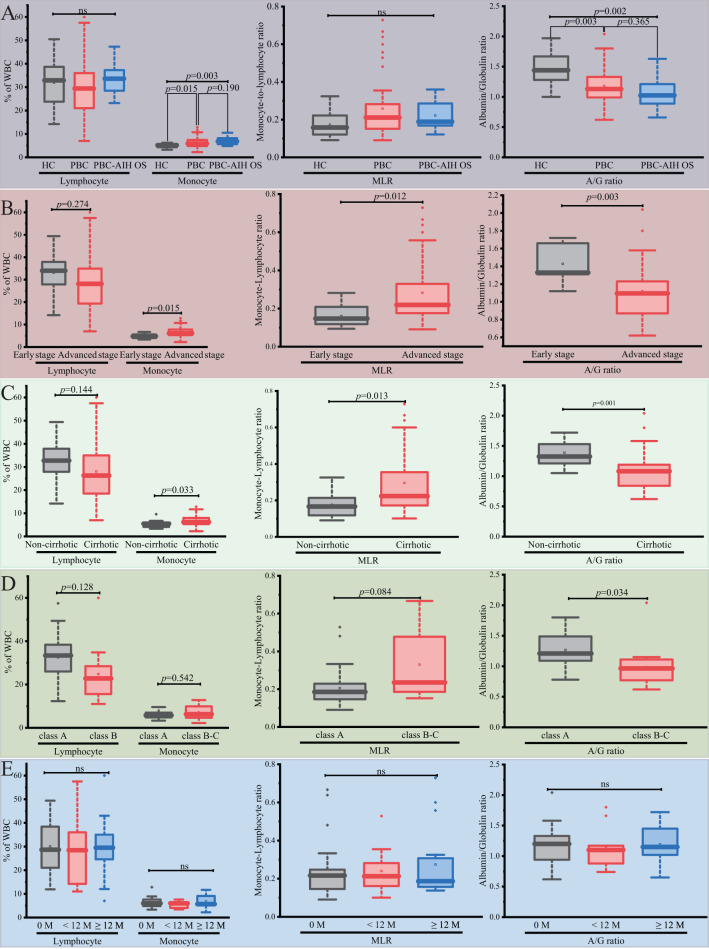
Comparative analysis of circulating monocyte counts, lymphocyte counts, MLR, and A/G ratio across study populations. (A) Comparison among HC and PBC patients and PBC-AIH OS patients. (B-D) PBC patient subgroups stratified by: (B) histological stage (early vs. advanced), (C) cirrhosis status (non-cirrhotic vs. cirrhotic), and (D) Child-Pugh classification (class A vs. B-C) in cirrhotic PBC patients. (E) PBC patients categorized by ursodeoxycholic acid treatment duration. A/G ratio, albumin/globulin ratio; HC, healthy control; M, months; MLR, monocyte-lymphocyte ratio; ns, no difference overall; PBC, primary biliary cholangitis; PBC-AIH OS, PBC-autoimmune hepatitis overlap syndrome; WBC, white blood cell.

Both PBC and PBC-AIH OS patients exhibited elevated monocyte percentages and reduced A/G ratio vs. HCs (Table 1, Figure 2A). Stratification by disease severity revealed that monocyte elevation (percentage count and MLR) and A/G ratio reduction were most pronounced in cirrhotic and advanced-stage patients (Figure 2B and C). Lymphocyte counts showed non-significant downward trends. Among cirrhotic patients, Child-Pugh class B-C demonstrated significantly lower A/G ratios but comparable monocyte, lymphocyte, and MLR values vs. class A (Figure 2D). Treatment status (naïve vs. UDCA-treated) did not influence these parameters (Figure 2E).

### High Serum Soluble Tim-3 and Low Galectin-9 Levels in Primary Biliary Cholangitis Patients

As shown in Table 2, PBC patients exhibited elevated sTim-3 and reduced Galectin-9 levels vs. controls. Neither biomarker differed significantly between cirrhotic and non-cirrhotic patients (Table 3), across disease stages (Supplementary Table 1), or by treatment duration (Supplementary Table 2). However, Child-Pugh B-C cirrhotics showed significantly lower Galectin-9 levels (Supplementary Table 3), suggesting these biomarkers may reflect disease state without correlating with progression.

### Abnormal Serum Levels of Cytokines and Chemokines in Patients with Primary Biliary Cholangitis

Univariate analysis revealed significant elevations in TNF-α, IL-10, CCL19, CXCL9, CXCL10, and CXCL11 in PBC/PBC-AIH OS vs. controls (Table 2). The CXCL10/11 levels were higher in PBC-AIH OS than in PBC, while CCL20 was specifically elevated in PBC patients. Conversely, CCL2 and CXCL16 levels were decreased in PBC.

Cirrhotic patients showed marked increases in TNF-α, IFN-γ, IL-6, IL-8, IL-10, CCL3, CCL19, CCL20, CXCL9-11, and CX3CL1, with trending CCL2 reduction (Table 3). Advanced-stage patients demonstrated similar patterns except for IL-8, CCL2, CCL19, and CXCL9 (Supplementary Table 1). Child-Pugh B-C patients exhibited particularly elevatedIL-6 and reduced CCL2 levels vs. class A (Supplementary Table 3). UDCA treatment duration did not significantly influence cytokine/chemokine profiles (Supplementary Table 2).

### Relationship Between Serum sTim-3, Galectin-9, Cytokines/Chemokines, and Clinical Parameters in Primary Biliary Cholangitis Patients

**Figure 3.**
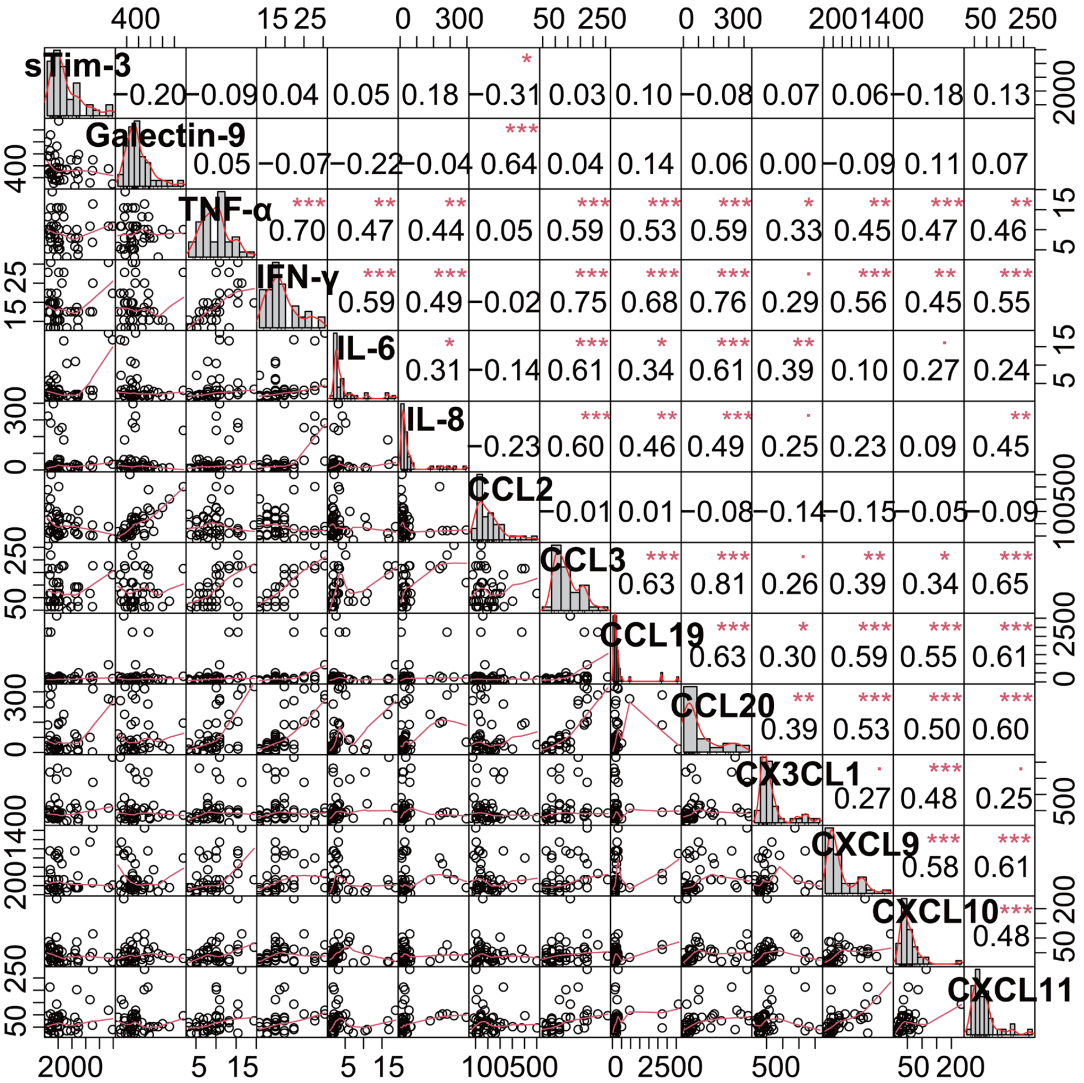
Correlation matrix of serum sTim-3, Galectin-9, cytokines, and chemokines in PBC patients. CCL, chemokine (C-C motif) ligand; CXCL, chemokine (C-X-C motif) ligand; CX3CL1, chemokine (C-X3-C motif) ligand 1; IFN-γ, interferon-gamma; IL, interleukin; sTim-3, soluble T-cell immunoglobulin and mucin-domain containing-3; TNF-α, tumor necrosis factor-alpha.

Spearman correlation analysis revealed distinct immunological patterns in PBC patients (Figure 3, and Table 4). The sTim-3 demonstrated only a weakly negative correlation with CCL2 (Spearman’s *r* = −0.311, *P* = .038) and showed no significant correlations with Galectin-9 or other parameters. In contrast, Galectin-9 exhibited positive correlations with CCL2 (*r* = 0.61, *P* < .001), ALB (*r* = 0.38, *P* = .01), and A/G ratio (*r* = .33, *P* = .027), while displaying a negative correlation with MLR (*r* = −0.47, *P* = .001). However, similar to sTim-3, Galectin-9 showed no association with liver disease severity markers (FIB-4, MELD, MELD-Na, or MRS1994).

The analysis identified several key immunological networks: The analysis identified several key immunological networks: TNF-α and IFN-γ showed strong intercorrelation (*r* = 0.70, *P* < .001) and collectively demonstrated significant positive associations with disease severity scores (MELD: *r* = 0.61-0.63; MRS1994: *r* = 0.63-0.64, all *P* < .001). The IL-6 emerged as the strongest predictor of MRS1994 (*r* = 0.76, *P* < .0001) while exhibiting inverse relationships with albumin (*r* = −0.71) and A/G ratio (*r* = −0.5). The CCL3 and CCL20 formed a tightly correlated pair (*r* = 0.81) with robust links to bilirubin levels (TBIL: *r* = 0.65-0.63) and prognostic scores (MELD-Na: *r* = 0.68-0.69). The CXCR3 chemokine ligands (CXCL9-11) demonstrated significant intra-group correlations (*r* = 0.48-0.61), with CXCL11 showing particular association with MELD-Na (*r* = 0.51, *P* < .001). The CX3CL1 displayed dual significance as both a fibrosis marker (FIB-4: *r* = 0.7, *P* < .0001) and a prognostic indicator (MRS1994: *r* = 0.61). In contrast, CCL2 exhibited inverse correlations with disease severity scores (MELD: *r* = −0.34, *P* = .023) but positive associations with albumin parameters (A/G ratio: *r* = 0.4, *P* = .007). These findings collectively delineate distinct immunological pathways associated with PBC progression.

### Potential Prognostic Factors for Primary Biliary Cholangitis Patients

From the initial correlation analysis, 10 variables showing associations with MRS1994 were selected for univariate linear regression analysis (Supplementary Table 4). Gender (non-significant, *P* > .05) and cirrhosis status were additionally included in the model. The LASSO regression analysis with 10-fold cross-validation identified 9 variables with non-zero coefficients (Supplementary Figure 1A). The optimal regularization parameter λ was determined to be 0.24 using the 1-standard error rule (Supplementary Figure 1B). The selected predictors included: cirrhosis, MLR, A/G ratio, IL-6, TNF-α, IFN-γ, CCL20, CX3CL1, and CXCL11. Covariance analysis confirmed the overall significance of this model (*F* = 23.817, *P* < .001).

In the final multivariable linear regression model (Table 5), 3 cytokines emerged as independent predictors of MRS1994: IL-6 (*β* = 1.592, 95% CI: 1.114-2.070, *P* < .001), TNF-α (*β* = 0.168, 95% CI: 0.076-0.259, *P* = .001), and CX3CL1 (*β* = 0.940, 95% CI: 0.4191.460, *P* = .001). When cytokines and chemokines were removed from the model, 3 clinical parameters retained significant predictive value: MLR (*β* = 1.339, 95% CI: 0.371-2.308, *P* = .008), A/G ratio (*β* = −1.725, 95% CI: −3.385 to −0.066, *P* = .042), and cirrhosis status (*β* = 1.436, 95% CI: 0.184-2.688, *P* = .026) (Supplementary Table 5).

## Discussion

The study demonstrated significantly elevated serum sTim-3 levels and reduced Galectin-9 concentrations in PBC patients compared with HCs, though neither biomarker showed significant clinical correlations with disease parameters. Serum IL-6, TNF-α, IFN-γ, CCL3, and CCL20 exhibited significant correlations with MELD and MELD-Na scores, while IL-6 and CX3CL1 showed strong correlations with the fibrosis-4 index. Furthermore, the data identified MLR, A/G ratio, cirrhosis status, TNF-α, IL-6, and CX3CL1 as potential predictors of PBC severity and outcome.

While sTim-3 has been previously associated with PBC disease course,^10^ upregulated serum sTim-3 and downregulated Galectin-9 levels were observed in PBC patients without significant relationships to clinical indicators, cytokines, or chemokines. The substantial proportion of UDCA-treated patients in the cohort may have influenced the assessment of sTim-3’s relationship with disease stage, severity, and prognosis. This finding aligns with studies reporting no correlation between serum sTim-3/Galectin-9 levels and liver fibrosis in AIH, suggesting Galectin-9 may not reflect liver function.^20^^,^^21^ Although sTim-3 functions as a pro-inflammatory regulator,^10^ its precise role in PBC pathogenesis require further investigation.

The MLR^22^^,^^23^ and A/G ratio^24^ have emerged as a novel inflammatory and prognostic biomarkers across various disease. In this study, both MLR and A/G ratio demonstrated potential prognostic value for PBC patients in the multiple linear regression model. These cost-effective biomarkers, readily available through routine clinical testing, warrant further evaluation in prospective PBC studies to confirm their prognostic utility.

Substantial evidence highlights marked upregulation of cytokines and chemokines in PBC diagnosis, UDCA non-response, and poor prognosis.^25^^-^^27^ The findings corroborate TNF-α, IL-6, and CX3CL1 as potential predictors of PBC severity and outcomes. However, biological therapies targeting multiple cytokines/chemokines have shown limited efficacy in PBC.^28^ Senescent biliary epithelial cells secrete various inflammatory factors (CCL2, CCL20, CX3CL1) that exacerbate PBC inflammation,^29^^,^^25^^,^^30^ with elevated chemokine levels potentially heralding the bile duct epithelial cell senescence process.^25^ The observation of increased CXCR3 ligands (CXCL9-11) in PBC patients supports previous reports,^30^^,^^31^ suggesting a dominant for CXCR3 axis in PBC pathogenesis. Contrary to expectations, decreased levels of the typical inflammatory chemokine CCL2 were found in PBC patients, consistent with 1 study showing no serum CCL2 differences between PBC patients and HCs,^31^ warranting further investigation of CCL2 expression in both serum and liver tissue.

The UDCA treatment modulates PBC immune responses by reducing pro-inflammatory cytokines,^28^ attenuating T-cell chemotaxis,^29^ and decreasing circulating CD19^+^ B cells.^32^ While partial biochemical improvement was observed with UDCA therapy, no significant changes occurred in circulating monocytes or serum levels of sTim-3, Galectin-9, cytokines, and chemokines among PBC patients. This may reflect suboptimal UDCA responses in some patients,^25^ or limitations imposed by the study’s retrospective design and modest sample size. Notably, while existing evidence indicates that UDCA therapy reduces IFN-γ and CX3CL1 levels,^29^ the relationship between immune checkpoint molecules and UDCA treatment response remains unexplored in PBC. Further studies are needed to determine whether dynamic changes in these immunologic parameters could serve as biomarkers for UDCA responsiveness.

Several study limitations merit consideration. First, the relatively small sample size may constrain the generalizability of ourthe findings, particularly regarding population-specific characteristics. Certain intergroup differences (e.g., FIB-4 scores between PBC and PBC-AIH overlap patients) lacked statistical power for definitive demonstration. While the cohort size may be insufficient for robust prognostic factor determination, the findings provide valuable preliminary insights for future research. Second, the absence of longitudinal follow-up precluded application of more refined prognostic models (e.g., UK-PBC and GLOBE scores) to assess treatment response and long-term outcomes. Although MRS1994—a validated predictor of liver transplantation or death in PBC—was utilized, the lack of survival data in the cohort necessitates further validation of its prognostic utility in clinical practice.

The findings demonstrate that while patients with PBC exhibit significantly elevated serum sTim-3 levels and reduced circulating Galectin-9 concentrations, these immune checkpoint molecules show no significant association with cytokine/chemokine profiles or clinical disease parameters. Notably, TNF-α, IL-6, CX3CL1, MLR, A/G ratio, and cirrhosis status emerge as promising biomarkers for assessing disease severity and predicting clinical outcomes in PBC. These results highlight the complex immunopathology of PBC and suggest potential targets for future prognostic evaluation and therapeutic investigation.

## Supplementary Materials

Supplementary Material

## Figures and Tables

**Table 1.** Characteristics of Study Subjects

	HC (n *=*22)	PBC (n = 45)	PBC-AIH OS (n = 10)	*P* (Overall)
Age, years	51 (6)	57 (10)	51 (16)	.057
Age at PBC diagnosis, years	–	55.1 (10.6)	48.0 (13.1)	
Female, n (%)	22 (100)	39 (86.7)	8 (80.0)	.090
AMA-M2-positive, n (%)	–	36 (83.7)	8 (80.0)	
Anti-gp210- positive, n (%)	–	16 (37.2)	6 (60.0)	
Anti-sp100-positive, n (%)	–	3 (7.14)	1 (10.0)	
Cirrhosis, n (%)	–	31 (68.9)	5 (50.0)	
Child-Pugh classification				
Class A, n (%)	–	31 (68.9)	8 (80.0)	
Class B, n (%)	–	12 (26.7)	2 (20.0)	
Class C, n (%)	–	2 (4.44)	0 (0.00)	
Histological stage				
Early, n (%)	–	9 (20.0)	4 (40.0)	
Advanced, n (%)	–	36 (80.0)	6 (60.0)	
Duration of UDCA treatment				
0 months, n (%)	–	18 (40.0)	2 (20.0)	
<12 months, n (%)	–	10 (22.2)	2 (20.0)	
≥12 months, n (%)	–	17 (37.8)	6 (60.0)	
Lymphocyte, %	31.9 (9.86)	29.6 (11.8)	33.7 (8.00)	.482
Monocyte, %, median (IQR)	5.20 [4.35; 5.65]	5.90 [4.80; 7.40]*	6.80 [5.82; 7.97]**	.004
Platelet count, 10^9^/L	274 (45.9)	166 (89.3)***	199 (88.3)	<.001
PT, s, median (IQR)	12.7 [12.3; 13.5]	12.9 [12.3; 14.2]	12.6 [11.7; 13.4]	.297
INR, median (IQR)	1.02 [0.95; 1.10]	1.00 [0.93; 1.12]	0.96 [0.89; 1.04]	.413
A/G ratio	1.46 (0.24)	1.18 (0.32)**	1.04 (0.29)**	.001
ALB, g/L	43.2 [39.9; 44.7]	36.5 [30.5; 41.6]***	37.5 [36.3; 42.1]*	<.001
ALT, IU/L, median (IQR)	17.0 [15.0; 24.0]	39.0 [19.0; 64.0]**	43.5 [31.8; 80.8]**	.001
AST, IU/L, median (IQR)	20.0 [16.5; 23.0]	44.0 [27.0; 77.0]***	45.5 [28.2; 68.0]***	<.001
ALP, IU/L, median (IQR)	81.5 [72.2; 91.2]	152 [101; 270]***	132 [109; 260] **	<.001
GGT, IU/L, median (IQR)	36 [31; 42]	90 [42; 178]	130 [33; 280]	.003
TBIL, μmol/L, median (IQR)	12.8 [9.40; 14.5]	20.0 [12.8; 45.5]**	15.6 [9.40; 23.0]	.001
Fibrosis-4 score, median (IQR)	–	2.77 [1.82; 5.28]	1.67 [1.13; 3.18]	
MELD, median (IQR)	–	7.80 [6.54; 12.0]	6.97 [6.45; 9.40]	
MELD-Na, median (IQR)	–	9.30 [7.08; 12.9]	7.65 [7.29; 9.63]	
MRS1994, median (IQR)	–	6.44 [5.10; 7.82]	5.16 [4.11; 6.19]	

*P < .05, **P < .01, ***P < .001. The PBC and PBC-AIH OS groups showed no significant differences.

A/G ratio, albumin/globulin ratio; ALB, albumin; ALP, alkaline phosphatase; ALT, alanine transaminase; AMA-M2, anti-mitochondrial antibody M2; AST, aspartate aminotransferase; GGT, gamma-glutamyl transferase; HC, healthy controls; INR, international normalized ratio; IQR, interquartile range; MELD, model for end-stage liver disease; MRS1994, Mayo risk score 1994; PBC, primary biliary cholangitis; PBC-AIH OS, PBC-autoimmune hepatitis overlap syndrome; PT, prothrombin time; TBIL, total bilirubin; UDCA, ursodeoxycholic acid.

**Table 2.** Serum Concentrations of sTim-3, Galectin-9, Cytokines, and Chemokines Across the Study Groups

	HC (n = 22)	PBC (n = 45)	PBC-AIH OS (n = 10)	*P* (Overall)
sTim-3, pg/mL, median (IQR)	1175.31 [874.78; 1772.48]	2070.64 [1149.97; 3834.94]*	1626.02 [1416.99; 1883.62]	.014
Galectin-9, pg/mL, median (IQR)	791.00 [646.50;1065.00]	518.00 [454.00; 694.00]***	555.00 [452.00; 742.50]	<.001
TNF-α, pg/mL, median (IQR)	6.86 [5.36; 7.94]	8.92 [6.86; 10.89]*	12.07 [9.20; 17.03]*	.003
IFN-γ, pg/mL, median (IQR)	16.43 [15.34; 19.55]	17.52 [15.34; 19.85]	21.10 [17.52; 24.34]	.115
IL-1β, pg/mL, median (IQR)	3.93 [3.93; 4.26]	3.93 [3.93; 4.37]	3.93 [3.93; 4.26]	.694
IL-2, pg/mL, median (IQR)	7.84 [6.98; 8.36]	7.84 [6.40; 9.49]	7.84 [6.46; 9.95]	.917
IL-6, pg/mL, median (IQR)	1.94 [1.75; 2.00]	2.00 [1.75; 3.44]	2.39 [2.06; 3.59]	.147
IL-8, pg/mL, median (IQR)	22.01 [14.70; 37.80]	20.71 [10.98; 42.60]	27.79 [13.28; 53.02]	.941
IL-10, pg/mL, median (IQR)	1.17 [1.06; 1.27]	1.94 [1.17; 2.49]***	2.21 [1.61; 6.12]***	<.001
IL-12, pg/mL, median (IQR)	156.32 [138.44; 156.32]	156.32 [135.93; 178.04]	156.32 [156.32; 172.61]	.464
CCL2, pg/mL, median (IQR)	301.74 [258.52; 340.97]	178.74 [121.99; 250.87]***	163.88 [130.97; 310.30]	.001
CCL3, pg/mL, median (IQR)	116.46 [85.69; 142.16]	116.46 [85.69; 159.65]	142.16 [116.46; 173.57]	.401
CCL4, pg/mL, median (IQR)	251.73 [207.43; 284.65]	237.70 [207.43; 349.76]	285.32 [237.70; 451.53]	.318
CCL19, pg/mL, median (IQR)	63.70 [52.73; 81.36]	145.17 [94.51; 251.44]***	185.28 [150.99; 241.11]***	<.001
CCL20, pg/mL, median (IQR)	38.03 [20.73; 52.67]	55.45 [22.71; 181.26]	63.66 [55.23; 97.71]*	.023
CXCL9, pg/mL, median (IQR)	309.80 [267.77; 354.59]	411.43 [354.59660.97]**	568.92 [460.49; 985.89]**	<.001
CXCL10, pg/mL, median (IQR)	17.59 [12.97; 22.09]	34.65 [24.37; 57.50]***	70.79 [44.54; 96.66]***, ^##^	<.001
CXCL11, pg/mL, median (IQR)	35.46 [25.87; 56.37]	64.05 [40.29; 90.28]**	105.38 [83.65; 219.48]**, ^##^	<.001
CXCL16, pg/mL	917.58 (153.57)	775.25 (217.10)*	770.05 (230.41)	.024
CX3CL1, pg/mL, median (IQR)	380.49 [310.48; 482.47]	402.60 [283.22; 578.37]	426.15 [361.91; 578.37]	.657

Compared with HC, *P < .05, **P < .01, ***P < .001; Compared with PBC patients, ^##^*P* < .01.

CCL, chemokine (C-C motif) ligand; CXCL, chemokine (C-X-C motif) ligand; CX3CL1, chemokine (C-X3-C motif) ligand 1; HC, healthy controls; IFN-γ, interferon-gamma; IL, interleukin; PBC, primary biliary cholangitis; PBC-AIH OS, PBC-autoimmune hepatitis overlap syndrome; sTim-3, soluble T-cell immunoglobulin and mucin-domain containing-3; TNF-α, tumor necrosis factor-alpha

**Table 3.** Comparative Analysis of Serum sTim-3, Galectin-9, Cytokines, and Chemokines in PBC Patients Stratified by Cirrhosis Status

	Non-cirrhotic (n = 14)	Cirrhotic (n = 31)	*P* (Overall)
sTim-3, pg/mL, median (IQR)	1835.97 [995.62; 2982.19]	2070.64 [1329.78; 4127.78]	.433
Galectin-9, pg/mL, median (IQR)	547.00 [491.00; 733.50]	498.00 [371.00; 634.00]	.145
TNF-α, pg/mL	7.68 (2.98)	10.17 (3.84)	.024
IFN-γ, pg/mL, median (IQR)	15.34 [13.32; 17.52]	18.66 [17.52; 22.35]	.003
IL-1β, pg/mL, median (IQR)	3.93 [3.93; 3.93]	4.37 [3.93; 4.82]	.122
IL-2, pg/mL, median (IQR)	6.86 [6.40; 7.71]	7.84 [6.86; 10.42]	.076
IL-6, pg/mL, median (IQR)	1.75 [1.52; 1.75]	3.16 [2.00; 5.53]	<.001
IL-8, pg/mL, median (IQR)	11.46 [9.02; 21.53]	28.70 [13.84; 60.08]	.014
IL-10, pg/mL, median (IQR)	1.31 [1.17; 1.57]	2.12 [1.69; 3.10]	.007
IL-12, pg/mL, median (IQR)	135.93 [135.93; 156.32]	156.32 [135.93; 178.04]	.094
CCL2, pg/mL, median (IQR)	238.36 [202.89; 296.66]	143.60 [115.91; 210.61]	.012
CCL3, pg/mL, median (IQR)	85.69 [63.92; 116.46]	116.46 [85.69; 215.35]	.007
CCL4, pg/mL, median (IQR)	222.56 [207.43; 343.96]	265.76 [207.43; 359.00]	.730
CCL19, pg/mL, median (IQR)	93.74 [68.24; 133.33]	162.30 [120.47; 264.88]	.008
CCL20, pg/mL, median (IQR)	29.16 [18.81; 44.75]	86.64 [41.42; 199.28]	.002
CXCL9, pg/mL, median (IQR)	354.59 [321.00; 397.22]	526.58 [354.59; 936.68]	.031
CXCL10, pg/mL, median (IQR)	25.92 [18.01; 33.25]	50.12 [32.49; 65.75]	.001
CXCL11, pg/mL, median (IQR)	49.57 [25.87; 68.60]	76.04 [49.57; 96.69]	.040
CXCL16, pg/mL, median (IQR)	720.37 [619.92; 885.15]	771.57 [622.78; 892.81]	.632
CX3CL1, pg/mL, median (IQR)	283.22 [254.11; 314.92]	449.71 [380.95; 686.45]	.002

CCL, chemokine (C-C motif) ligand; CXCL, chemokine (C-X-C motif) ligand; CX3CL1, chemokine (C-X3-C motif) ligand 1; IFN-γ, interferon-gamma; IL, interleukin; PBC, primary biliary cholangitis; sTim-3, soluble T-cell immunoglobulin and mucin-domain containing-3; TNF-α, tumor necrosis factor-alpha.

**Table 4.** Spearman Correlation Analysis of Clinical Parameters with Serum sTim-3, Galectin-9, Cytokines, and Chemokines in PBC Patients

		Monocyte	ALB	A/G ratio	ALP	TBIL	MLR	Fibrosis-4	MELD	MELD-Na	MRS1994
sTim-3	*r*	0.05	−0.22	−0.26	0.03	0.01	0.13	0.21	−0.03	0.03	0.08
	*P*	.765	.140	.089	.867	.946	.392	.158	.849	.847	.595
Galectin-9	*r*	−0.17	0.38	0.33	0.02	−0.02	−0.47	−0.28	−0.1	−0.12	−0.22
	*P*	.275	.010	.027	.899	.884	.001	.067	.499	.432	.152
TNF-α	*r*	0.16	−0.3	−0.24	0.25	0.62	0.2	0.41	0.61	0.56	0.63
	*P*	.302	.044	.107	.097	<.000	.184	.005	<.000	<.000	<.000
IFN-γ	*r*	0.26	−0.4	−0.4	0.4	0.67	0.42	0.44	0.63	0.65	0.64
	*P*	.091	.006	.007	.006	<.000	.004	.003	<.000	<.000	<.000
IL-6	*r*	0.14	−0.71	−0.5	0.04	0.47	0.37	0.6	0.63	0.6	0.76
	*P*	.351	<.000	.001	.793	.001	.012	<.000	<.000	<.000	<.000
IL-8	*r*	0.25	−0.24	−0.5	0.32	0.58	0.42	0.25	0.45	0.51	0.39
	*P*	.094	.115	.001	.031	<.000	.004	.096	.002	.000	.008
CCL2	*r*	0	0.39	0.4	−0.21	−0.2	−0.31	−0.34	−0.29	−0.32	−0.28
	*P*	.977	.008	.007	.165	.203	.035	.023	.055	.031	.066
CCL3	*r*	0.08	−0.41	−0.33	0.23	0.65	0.32	0.3	0.61	0.68	0.58
	*P*	.593	.005	.026	.129	<.000	.033	.044	<.000	<.000	<.000
CCL19	*r*	0.19	−0.26	−0.38	0.42	0.38	0.27	0.33	0.3	0.38	0.34
	*P*	.218	.087	.009	.004	.010	.072	.025	.048	.009	.021
CCL20	*r*	0.16	−0.41	−0.4	0.36	0.63	0.4	0.44	0.66	0.69	0.65
	*P*	.283	.005	.006	.014	<.000	.006	.002	<.000	<.000	<.000
CXCL9	*r*	0.07	−0.17	−0.36	0.53	0.3	0.27	0.26	0.27	0.3	0.31
	*P*	.626	.267	.015	.000	.501	.074	.083	.073	.047	.042
CXCL10	*r*	0.08	−0.22	−0.32	0.33	0.34	0.03	0.49	0.35	0.36	0.4
	*P*	.623	.152	.033	.025	.024	.857	.001	.018	.015	.007
CXCL11	*r*	0.09	−0.24	−0.36	0.5	0.44	0.13	0.25	0.41	0.51	0.43
	*P*	.552	.116	.014	.000	.003	.400	.09	.004	.000	.003
CX3CL1	*r*	0.21	−0.49	−0.29	0.27	0.54	0.37	0.7	0.58	0.57	0.61
	*P*	.169	.001	.051	.075	.000	<.000	<.000	<.000	<.000	<.000

A/G ratio, albumin/globulin ratio; ALB, albumin; ALP, alkaline phosphatase; CCL, chemokine (C-C motif) ligand; CXCL, chemokine (C-X-C motif) ligand; CX3CL1, chemokine (C-X3-C motif) ligand 1; IFN-γ, interferon-gamma; IL-6: interleukin-6; IL-8: interleukin-8; MELD, model for end-stage liver disease; PBC, primary biliary cholangitis; MLR, monocyte-lymphocyte ratio; MRS1994, Mayo risk score 1994; r, Spearman correlation coefficient; sTim-3, soluble T-cell immunoglobulin and mucin-domain containing-3; TBIL, total bilirubin; TNF-α, tumor necrosis factor-alpha.

**Table 5.** Multivariable Linear Regression Model Identifying Prognostic Factors for PBC Severity (MRS1994)

Predictors	MRS1994		
	Estimates	95% CI	*P*
IL-6*	1.592	1.114-2.070	**<.001**
CX3CL1*	0.940	0.419-1.460	**.001**
TNF-α	0.168	0.076-0.259	**.001**

R2/R2 adjusted: 0.757/0.739.

*Transformed by a natural log conversion.

CX3CL1, chemokine (C-X3-C motif) ligand 1; IL-6, interleukin-6; MRS1994, Mayo risk score 1994; PBC, primary biliary cholangitis; TNF-α, Tumor necrosis factor alpha.

## Data Availability

The data that support the findings of this study are available on request from the corresponding author.
