# Supplementary material for: Elevated Serum Soluble Tim-3 in Primary Biliary Cholangitis: Lack of Correlation with Cytokines, Chemokines, and Clinical Parameters
Source: Turk J Gastroenterol. 2025 Aug 25;37(2):196–207. doi: 10.5152/tjg.2025.24520 (PMC12910306; doi:10.5152/tjg.2025.24520)
Supplement: Supplementary Material [file supplementary_material.pdf]

Supplementary Table 1. Serum biomarker profiles in PBC patients categorized by disease stage (early vs. advanced)

	Early stage *N=9*	Advanced stage N=36	*P.Overall*
sTim-3, pg/mL, median (IQR)	2116.65 [1504.23;4871.27]	1956.63 [1115.06;3409.69]	.843
Galectin-9, pg/mL, median (IQR)	564.00 [506.00;778.00]	502.00 [406.00;665.50]	.160
TNF-α, pg/mL	7.02 (2.33)	9.99 (3.82)	.008
IFN-γ, pg/mL, median (IQR)	15.34 [13.32;15.34]	19.26 [17.52;22.35]	.002
IL-1β, pg/mL, median (IQR)	3.93 [3.93;4.37]	3.93 [3.93;4.82]	.654
IL-2, pg/mL, median (IQR)	6.86 [6.86;7.33]	7.84 [6.40;9.64]	.215
IL-6, pg/mL, median (IQR)	1.52 [1.52;1.75]	2.68 [1.94;4.46]	<.001
IL-8, pg/mL, median (IQR)	11.76 [8.20;21.81]	27.73 [12.40;55.84]	.052
IL-10, pg/mL, median (IQR)	1.31 [1.17;1.45]	2.12 [1.42;3.02]	.012
IL-12, pg/mL, median (IQR)	135.93 [135.93;156.32]	156.32 [135.93;178.04]	.347
CCL2, pg/mL, median (IQR)	232.22 [200.69;261.87]	153.90 [121.21;242.96]	.222
CCL3, pg/mL, median (IQR)	85.69 [63.92;116.46]	116.46 [85.69;197.06]	.035
CCL4, pg/mL, median (IQR)	207.43 [177.54;284.65]	265.76 [207.43;368.25]	.236
CCL19, pg/mL, median (IQR)	71.82 [49.23;145.17]	155.51 [104.39;252.53]	.054
CCL20, pg/mL, median (IQR)	19.80 [11.27;35.49]	77.57 [39.41;196.72]	.002
CXCL9, pg/mL, median (IQR)	354.59 [309.80;411.43]	493.54 [354.59;905.15]	.057
CXCL10, pg/mL, median (IQR)	24.37 [17.29;34.35]	47.20 [30.73;62.24]	.008
CXCL11, pg/mL, median (IQR)	25.87 [25.87;64.05]	68.60 [47.02;96.69]	.013
CXCL16, pg/mL	758.44 [720.37;885.15]	745.53 [614.62;888.98]	.898
CX3CL1, pg/mL, median (IQR)	283.22 [250.06;300.98]	449.71 [349.36;673.92]	.002

PBC, primary biliary cholangitis; sTim-3, soluble T cell immunoglobulin and mucin domain 3; TNF-α, Tumor necrosis factor alpha; IFN-γ, Interferon-gamma; IL, Interleukin; CCL, Chemokine (C-C motif) ligand; CX3CL1, chemokine (C-X3-C motif) ligand 1; CXCL, Chemokine (C-X-C motif) ligand.

Supplementary Table 2. Impact of UDCA treatment duration on serum sTim-3, Galectin-9, cytokines, and chemokines in PBC patients

	0 months *N=18*	< 12 months *N=10*	≥ 12 months *N=17*	*P*.Overall
sTim-3, pg/mL, median (IQR)	2030.64 [1517.67;2212.38]	2952.79 [1821.76;4481.54]	1743.78 [990.71;4885.07]	.649
Galectin-9, pg/mL, median (IQR)	547.00 [484.50;772.50]	491.00 [460.00;697.00]	486.00 [376.00;550.00]	.197
TNF-α, pg/mL	9.17 (3.84)	9.88 (4.05)	9.35 (3.68)	.893
IFN-γ, pg/mL, median (IQR)	17.52 [15.34;19.85]	18.09 [17.52;21.73]	17.52 [15.34;19.85]	.889
IL-1β, pg/mL, median (IQR)	3.93 [3.93;4.37]	4.15 [3.93;4.71]	3.93 [3.93;4.37]	.391
IL-2, pg/mL, median (IQR)	7.58 [6.08;8.77]	7.84 [7.33;10.58]	7.84 [6.86;9.49]	.655
IL-6, pg/mL, median (IQR)	1.88 [1.75;2.53]	2.26 [1.81;3.16]	3.16 [1.75;7.20]	.156
IL-8, pg/mL, median (IQR)	22.78 [9.82;60.39]	22.49 [14.45;51.89]	16.29 [11.17;31.28]	.728
IL-10, pg/mL, median (IQR)	2.12 [1.39;2.44]	2.21 [1.36;5.69]	1.45 [1.17;2.12]	.339
IL-12, pg/mL, median (IQR)	146.12 [135.93;178.04]	156.32 [135.93;178.04]	156.32 [135.93;156.32]	.734
CCL2, pg/mL, median (IQR)	205.09 [143.60;267.90]	141.93 [114.42;173.85]	188.99 [121.99;253.03]	.393
CCL3, pg/mL, median (IQR)	101.07 [85.69;183.14]	142.16 [116.46;159.65]	116.46 [85.69;116.46]	.573
CCL4, pg/mL, median (IQR)	237.70 [207.43;349.76]	285.32 [177.54;335.11]	237.70 [207.43;368.25]	.999
CCL19, pg/mL, median (IQR)	164.42 [100.47;268.34]	169.82 [104.19;306.34]	112.21 [76.92;145.17]	.138
CCL20, pg/mL, median (IQR)	51.80 [27.17;191.71]	61.80 [39.78;117.32]	51.77 [19.80;110.18]	.895
CXCL9, pg/mL, median (IQR)	526.58 [354.59;968.20]	411.43 [354.59;952.90]	354.59 [309.80;526.58]	.285
CXCL10, pg/mL, median (IQR)	36.70 [29.83;53.68]	53.55 [33.12;80.64]	29.95 [20.18;50.12]	.216
CXCL11, pg/mL, median (IQR)	75.02 [52.12;95.09]	71.20 [41.97;130.20]	47.02 [35.46;68.60]	.113
CXCL16, pg/mL, median (IQR)	792.42 [630.65;1002.37]	732.95 [607.02;859.16]	720.37 [617.25;885.15]	.598
CX3CL1, pg/mL, median (IQR)	380.95 [283.22;443.70]	547.56 [414.38;678.24]	402.60 [283.22;661.38]	.239

PBC, primary biliary cholangitis; UDCA, Ursodeoxycholic acid; sTim-3, soluble T cell immunoglobulin and mucin domain 3; TNF-α, Tumor necrosis factor alpha; IFN-γ, Interferon-gamma; IL, Interleukin; CCL, Chemokine (C-C motif) ligand; CX3CL1, chemokine (C-X3-C motif) ligand 1; CXCL, Chemokine (C-X-C motif) ligand.

Supplementary Table 3. Serum biomarker variations between Child-Pugh class A and B-C cirrhotic PBC patients

	class A *N=17*	class B-C *N=1*	*P*.Overall
sTim-3, pg/mL, median (IQR)	1922.62 [1314.13;2082.12]	2531.74 [1296.56;4440.93]	.352
Galectin-9, pg/mL, median (IQR)	544.00 [478.00;766.00]	371.00 [345.50;508.50]	.009
TNF-α, pg/mL	9.76 (3.52)	10.64 (4.65)	.585
IFN-γ, pg/mL, median (IQR)	17.52 [17.52;19.85]	18.69 [17.52;25.72]	.555
IL-1β, pg/mL, median (IQR)	3.93 [3.93;4.82]	4.37 [3.93;4.72]	.467
IL-2, pg/mL, median (IQR)	7.84 [5.97;10.10]	8.38 [7.84;10.74]	.463
IL-6, pg/mL, median (IQR)	2.53 [1.75;3.16]	3.84 [3.00;8.01]	.018
IL-8, pg/mL, median (IQR)	20.36 [13.49;55.11]	30.62 [13.44;91.65]	.690
IL-10, pg/mL, median (IQR)	2.12 [1.77;3.27]	1.94 [1.57;2.60]	.505
IL-12, pg/mL, median (IQR)	156.32 [135.93;178.04]	156.32 [135.93;167.51]	.891
CCL2, pg/mL, median (IQR)	159.18 [125.18;275.71]	128.61 [104.54;154.25]	.033
CCL3, pg/mL, median (IQR)	116.46 [85.69;159.65]	129.31 [85.69;226.73]	.272
CCL4, pg/mL, median (IQR)	237.70 [207.43;326.55]	251.73 [165.46;383.63]	.894
CCL19, pg/mL, median (IQR)	162.30 [140.27;222.97]	136.95 [103.56;280.11]	.535
CCL20, pg/mL, median (IQR)	68.14 [40.71;110.18]	110.77 [46.71;389.50]	.250
CXCL9, pg/mL, median (IQR)	526.58 [354.59;873.62]	526.58 [332.88;1081.25]	.738
CXCL10, pg/mL, median (IQR)	55.08 [33.77;69.45]	43.17 [22.67;61.84]	.223
CXCL11, pg/mL, median (IQR)	57.78 [52.12;90.28]	81.64 [49.16;98.41]	.549
CXCL16, pg/mL, median (IQR)	762.18 (152.00)	751.18 (254.39)	.895
CX3CL1, pg/mL, median (IQR)	449.71 [359.29;578.37]	437.70 [381.84;758.37]	.773

PBC, primary biliary cholangitis; sTim-3, soluble T cell immunoglobulin and mucin domain 3; TNF-α, Tumor necrosis factor alpha; IFN-γ, Interferon-gamma; IL, Interleukin; CCL, Chemokine (C-C motif) ligand; CX3CL1, chemokine (C-X3-C motif) ligand 1; CXCL, Chemokine (C-X-C motif) ligand.

Supplementary Table 4. Univariate linear regression of MRS1994-associated variables in PBC patients

	MRS1994		
Variables	*Estimates*	*CI*	*p*
Gender			
Female	0.54	-1.31 – 2.39	.561
Cirrhosis	2.56	1.41 – 3.71	<.001
MLR*	1.90	0.87 – 2.93	.001
A/G ratio	-2.77	-4.58 – -0.95	.004
IL-6*	2.11	1.53 – 2.68	<.001
CCL3	0.02	0.01 – 0.03	<.001
CCL20*	1.10	0.71 – 1.48	<.001
CX3CL1*	1.69	0.88 – 2.51	<.001
CXCL10*	1.04	0.08 – 2.01	.035
CXCL11*	1.44	0.50 – 2.37	.004
TNF-α	0.30	0.15 – 0.44	<.001
IFN-γ	5.66	3.56 – 7.75	<.001

*Transformed by a natural log conversion.

PBC, primary biliary cholangitis; CI, confidence interval; MRS1994, Mayo risk score 1994; MLR, Monocyte-Lymphocyte ratio; A/G ratio, Albumin/Globulin ratio; TNF-α, Tumor necrosis factor alpha; IFN-γ, Interferon-gamma; IL-6, Interleukin-6; CCL, Chemokine (C-C motif) ligand; CX3CL1, chemokine (C-X3-C motif) ligand 1; CXCL, Chemokine (C-X-C motif) ligand.

Supplementary Table 5. Adjusted multivariable linear regression model of prognostic factors (excluding cytokines/chemokines) for PBC patients

	MRS1994		
Predictors	*Estimates*	*95% CI*	*p*
Cirrhosis	1.436	0.184 – 2.688	**.026**
MLR*	1.339	0.371 – 2.308	**.008**
A/G ratio	-1.725	-3.385 – 0.066	**.042**

R^2 / R^2 adjusted: 0.472 / 0.433.

*Transformed by a natural log conversion.

PBC, primary biliary cholangitis; CI, confidence interval; MRS1994, Mayo risk score 1994; MLR, Monocyte-Lymphocyte ratio; A/G ratio, Albumin/Globulin ratio.

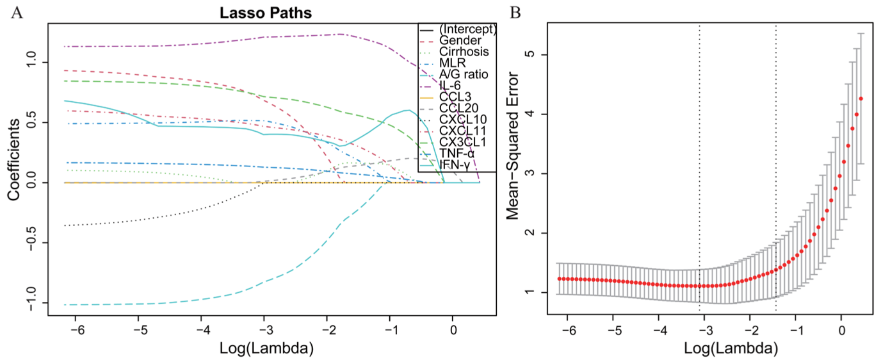

Supplementary Figure 1. Variable selection using LASSO-penalized linear regression modeling. (A) Coefficient profile plot showing variable selection against the log(λ) sequence. Nine variables with nonzero coefficients were retained at optimal λ. (B) Partial likelihood deviance curve with optimal λ selection using the 1 standard error criteria. MLR, Monocyte-Lymphocyte ratio; A/G ratio, Albumin/Globulin ratio; IL-6, Interleukin 6; CCL, Chemokine (C-C motif) ligand; CX3CL1, Fractalkine, chemokine (C-X3-C motif) ligand 1; CXCL, Chemokine (C-X-C motif) ligand; TNF-α, Tumor necrosis factor-alpha; IFN-γ, Interferon-gamma.
